# Venous thromboembolism in Japanese patients with breast cancer: subgroup analysis of the Cancer-VTE Registry

**DOI:** 10.1007/s12282-023-01452-7

**Published:** 2023-04-17

**Authors:** Shozo Ohsumi, Kenichi Watanabe, Naoto Kondo, Yoshimasa Kosaka, Takashi Ishikawa, Miyuki Kitahara, Shinichiro Kubo, Mari S. Oba, Tetsuya Kimura, Atsushi Takita, Hirofumi Mukai

**Affiliations:** 1 Department of Breast Oncology, National Hospital Organization Shikoku Cancer Center, 160 Kou, Minami-umemoto-machi, Matsuyama, Ehime 791-0280 Japan; 2 Department of Breast Surgery, National Hospital Organization Hokkaido Cancer Center, Sapporo, Hokkaido Japan; 3 Department of Breast Surgery, Nagoya City University Graduate School of Medical Sciences, Nagoya, Aichi Japan; 4 Department of Breast and Endocrine Surgery, Kitasato University School of Medicine, Sagamihara, Kanagawa Japan; 5 Department of Breast Surgery, Japanese Red Cross Sagamihara Hospital, Sagamihara, Kanagawa Japan; 6 Department of Breast Oncology and Surgery, Tokyo Medical University Hospital, Shinjuku-ku, Tokyo, Japan; 7 Department of Breast Surgery, Ibaraki Prefectural Central Hospital, Ibaraki Cancer Center, Kasama, Ibaraki Japan; 8 Division of Breast and Thyroid Gland Surgery, Fukuyama City Hospital, Fukuyama, Hiroshima Japan; 9 Department of Medical Statistics, Toho University, Ota-ku, Tokyo, Japan; 10 Department of Clinical Data Science, Clinical Research and Education Promotion Division, National Center of Neurology and Psychiatry, Kodaira, Tokyo Japan; 11 Primary Medical Science Department, Daiichi Sankyo Co., Ltd., Chuo-ku, Tokyo, Japan; 12 Data Intelligence Department, Daiichi Sankyo Co., Ltd., Shinagawa-ku, Tokyo, Japan; 13 Division of Medical Oncology, National Cancer Center Hospital East, Kashiwa, Chiba Japan

**Keywords:** Venous thromboembolism, Breast cancer, Japan

## Abstract

**Background:**

This subgroup analysis of the Cancer-VTE Registry, a nationwide, large-scale, multicenter observational study with a 1-year follow-up, assessed real-world data on venous thromboembolism (VTE) among Japanese patients with breast cancer.

**Methods:**

Patients with stage II–IV pretreatment breast cancer screened for VTE at enrollment were included. During the 1-year follow-up period, incidences of VTE, bleeding, and all-cause death, and background factors associated with VTE risk were examined.

**Results:**

Of 9,630 patients in the Cancer-VTE Registry analysis set, 993 (10.3%) had breast cancer (973 [98.0%] did not have and 20 [2.0%] had VTE at baseline). The mean age was 58.4 years, 73.4% of patients had stage II cancer, and 94.8% had an Eastern Cooperative Oncology Group performance status (ECOG PS) of 0. Risk factors for VTE at baseline by univariable analysis were age ≥ 65 years, ECOG PS of 2, VTE history, and D-dimer > 1.2 μg/mL. During follow-up, the incidence of symptomatic VTE was 0.4%; incidental VTE requiring treatment, 0.1%; composite VTE (symptomatic VTE and incidental VTE requiring treatment), 0.5%; bleeding, 0.2%; cerebral infarction/transient ischemic attack/systemic embolic event, 0.2%; and all-cause death, 2.1%. One patient with symptomatic VTE developed pulmonary embolism (PE) and died. Incidences of VTE and all-cause death were higher in patients with VTE vs without VTE at baseline.

**Conclusions:**

In Japanese patients with breast cancer, VTE screening before initiating cancer treatment revealed a 2.0% prevalence of VTE. During follow-up, one patient had a fatal outcome due to PE, but the incidences of VTE were low.

**Clinical trial registration:**

UMIN000024942; UMIN Clinical Trials Registry: https://www.umin.ac.jp/ctr/.

**Supplementary Information:**

The online version contains supplementary material available at 10.1007/s12282-023-01452-7.

## Introduction

Globally, breast cancer is the most frequently diagnosed cancer among women, with an estimated 2.3 million new cases in 2020 [1]. In contrast with women in Western countries, Japanese women tend to be at a lower risk of developing breast cancer [2], although breast cancer is still the leading cancer among Japanese women [1]. Venous thromboembolism (VTE) risk in cancer patients varies by ethnicity, patient, and treatment characteristics [3], and such variability has also been reported for breast cancer [4–7]. Although the absolute risk of VTE is relatively low for patients with breast cancer compared with other cancer populations [8], patients with breast cancer have a three- to four-fold increased risk of developing VTE compared with women without cancer [9].

In addition, because breast cancer is currently the most frequently occurring cancer among women [10], the absolute number of patients with breast cancer with VTE complications encountered in clinical practice is also likely to be high [11]. However, data on the prevalence and incidence of VTE and VTE-related complications among patients with breast cancer are scarce globally and in Japan. Therefore, the actual VTE status among patients with breast cancer needs to be investigated.

Breast cancer, depending on the subtype (i.e., hormone receptor positive [+]/human epidermal growth factor receptor 2 (HER2) negative, HER2+, and triple negative), is systemically treated with drug therapy and intensive radiation. Hormone therapy is the mainstay of drug therapy for hormone receptor positive breast cancer. Radiation and hormonal therapy have been reported as risk factors for VTE, but few prospective studies have analyzed these associations among patients with breast cancer [12–16].

The Cancer-VTE Registry, a nationwide, prospectively collected registry database including about 10,000 participants, aimed to evaluate the occurrence and management of VTE in Japanese patients with six major solid tumors (colorectal, lung, stomach, pancreatic, breast, and gynecologic cancer) [17]. The baseline data and main outcomes for the overall population have been reported [3, 18]. A marked difference in VTE incidence was observed by cancer type [3, 18], validating further analysis of the registry data by cancer type. The present subgroup analysis focuses on the prevalence of VTE at baseline, the incidence of VTE events and all-cause death, and the evaluation of background factors associated with VTE risk among patients with breast cancer enrolled in the Registry.

## Materials and methods

### Study design

The study rationale and design details have been previously reported [3, 18]. Briefly, the Cancer-VTE Registry was a nationwide, large-scale multicenter observational study in Japan undertaken between March 2017 and February 2019, with a 1-year follow-up.

The ethics committee at each participating institution approved the protocol. The study adhered to the Declaration of Helsinki and the Ethical Guidelines for Medical Science Studies on Human Subjects by the Japanese Ministry of Education, Culture, Sports, Science and Technology and the Ministry of Health, Labour and Welfare. All patients provided written informed consent.

### Patients

Enrolled patients included hospitalized patients or outpatients aged ≥ 20 years with a diagnosis of breast cancer, confirmed stage II–IV cancer with planned initiation of cancer therapy, Eastern Cooperative Oncology Group Performance Status (ECOG PS) of 0–2, and a life expectancy of ≥ 6 months. All patients had undergone VTE screening via lower extremity venous ultrasonography or computed tomography angiography 2 months before enrollment [19]. However, the VTE negative predictive value is extremely high at d-dimer values of 1.2 µg/mL or less [20]. Thus, if the D-dimer value measured after the cancer diagnosis was 1.2 μg/mL or less, VTE screening was not necessarily required, and the patient was considered to have no VTE. Patients were excluded if they had active double cancer, if their follow-up was difficult, or if participation in this study was deemed inappropriate by the investigator.

### Outcomes

The prevalence of VTE at baseline was analyzed, including symptomatic/incidental pulmonary embolism (PE) and symptomatic/asymptomatic deep vein thrombosis (DVT), both proximal and distal. Additionally, risk factors for VTE at baseline were analyzed. At follow-up, the cumulative incidences of symptomatic VTE, composite VTE (symptomatic VTE events and incidental [asymptomatic] VTE events requiring treatment), bleeding (major or clinically relevant non-major bleeding), cerebral infarction/transient ischemic attack (TIA)/systemic embolic event (SEE), and all-cause death were calculated.

### Statistical analysis

Details of the statistical analysis, including sample size calculations, have been reported [3]. Of the planned 10,000 participants, 1000 patients with breast cancer were estimated using the predicted numbers of cancer patients in Japan [17, 21]. Categorical variables were tabulated (n [%]), and continuous variables were calculated as mean and standard deviation (SD). Time-to-event rates were calculated using the cumulative incidence function for each event of interest. Between-group differences according to baseline VTE status were explored using the Gray test (for VTE, bleeding, and cerebral infarction/TIA/SEE) or the log-rank test (for all-cause death). Univariable analyses were conducted to detect risk factors for VTE at baseline using logistic regression models and risk factors for composite VTE during the follow-up periods using the Fine and Gray models, with all-cause death as a competing event. A two-sided *P* < 0.05 was considered statistically significant. The data analysis was conducted using SAS software version 9.4 (SAS Institute Inc., Cary, NC, USA).

## Results

### Prevalence of VTE at baseline

The total number of patients enrolled in the overall study was 10,202, of which 9,630 patients formed the Cancer-VTE Registry analysis set, and of these, 993 (10.3%) had breast cancer. Of the patients with breast cancer, 20 (2.0%) had VTE at baseline, all of whom had asymptomatic DVT (Table 1).

**Table 1:** Summary of VTE prevalence at baseline among patients with breast cancer (*n* = 993)

	Total	Symptomatic	Asymptomatic
All VTE, *n* (%)	20 (2.0)	0 (0.0)	20 (2.0)
PE (with/without DVT)	0 (0.0)	0 (0.0)	0 (0.0)
DVT (with/without PE)	20 (2.0)	0 (0.0)	20 (2.0)
Proximal DVT	1 (0.1)	0 (0.0)	1 (0.1)
Distal DVT	19 (1.9)	0 (0.0)	19 (1.9)

*DVT* deep vein thrombosis, *PE* pulmonary embolism, *VTE* venous thromboembolism

### Baseline characteristics

The mean age at baseline of the 993 patients with breast cancer was 58.4 years (Table 2). Most patients with breast cancer had stage II cancer (73.4%), ECOG PS of 0 (94.8%), and invasive type (95.7%). The number (percentage) of patients with each breast cancer type was as follows: non-invasive, 17 (1.7%); invasive, 950 (95.7%); invasive ductal, 876 (88.2%); estrogen receptor positive (ER+), 740 (74.5%); progestogen receptor positive (PgR+), 594 (59.8%); and HER2+, 251 (25.3%) breast cancer.

**Table 2:** Baseline demographic and clinical characteristics of patients with breast cancer in the Cancer-VTE Registry

	Patients with breast cancer (*n* = 993)	Patients with VTE at baseline (*n* = 20)	Patients without VTE at baseline (*n* = 973)
Female sex, *n* (%)	990 (99.7)	20 (100.0)	970 (99.7)
Age, years, mean ± SD	58.4 ± 13.2	68.0 ± 11.4	58.2 ± 13.2
BMI, kg/m^2^			
Mean ± SD	23.76 ± 4.41	24.56 ± 4.74	23.74 ± 4.41
≥ 25, *n* (%)	331 (33.3)	8 (40.0)	323 (33.2)
≥ 35, *n* (%)	25 (2.5)	1 (5.0)	24 (2.5)
Primary cancer, *n* (%)	938 (94.5)	18 (90.0)	920 (94.6)
Cancer stage, *n* (%)			
II	729 (73.4)	12 (60.0)	717 (73.7)
III	165 (16.6)	4 (20.0)	161 (16.5)
IV	99 (10.0)	4 (20.0)	95 (9.8)
ECOG PS, *n* (%)			
0	941 (94.8)	16 (80.0)	925 (95.1)
1	45 (4.5)	1 (5.0)	44 (4.5)
2	7 (0.7)	3 (15.0)	4 (0.4)
Cancer type, *n* (%)			
Non-invasive cancer	17 (1.7)	1 (5.0)	16 (1.6)
Invasive	950 (95.7)	18 (90.0)	932 (95.8)
Invasive ductal breast cancer	876 (88.2)	18 (90.0)	858 (88.2)
Other	74 (7.5)	0 (0.0)	74 (7.6)
ER, *n* (%)			
Positive	740 (74.5)	13 (65.0)	727 (74.7)
Negative	235 (23.7)	7 (35.0)	228 (23.4)
PgR, *n* (%)			
Positive	594 (59.8)	11 (55.0)	583 (59.9)
Negative	381 (38.4)	9 (45.0)	372 (38.2)
HER2, *n* (%)			
Positive^a^	251 (25.3)	5 (25.0)	246 (25.3)
Negative	711 (71.6)	15 (75.0)	696 (71.5)
D-dimer, μg/mL			
Mean ± SD	0.86 ± 3.81	2.86 ± 2.16	0.82 ± 3.83
> 1.2, *n* (%)	84 (8.5)	14 (70.0)	70 (7.2)
CrCl, mL/min			
Mean ± SD	91 ± 30	73 ± 28	92 ± 30
≤ 50, *n* (%)	59 (5.9)	3 (15.0)	56 (5.8)
Platelet count, × 10^9^/L			
Mean ± SD	261 ± 67	255 ± 101	261 ± 66
≥ 350, *n* (%)	87 (8.8)	2 (10.0)	85 (8.7)
Hemoglobin, g/dL			
Mean ± SD	13.3 ± 1.4	13.1 ± 1.5	13.3 ± 1.4
< 10, *n* (%)	23 (2.3)	0 (0.0)	23 (2.4)
WBC count, × 10^9^/L			
Mean ± SD	6.29 ± 1.73	6.06 ± 2.49	6.30 ± 1.71
> 11, *n* (%)	16 (1.6)	1 (5.0)	15 (1.5)

*BMI* body mass index, *CrCl* creatinine clearance, *ECOG PS* Eastern Cooperative Oncology Group Performance Status, *ER* estrogen receptor, *HER2* human epidermal growth factor receptor 2, *ICH* immunohistochemistry, *PgR* progesterone receptor, *SD* standard deviation, *VTE* venous thromboembolism, *WBC* white blood cell

^a^HER2 positive is a composite of ICH3 positive or ICH2 positive with ICH amplification

Descriptive comparisons showed that patients with VTE had a higher proportion of ECOG PS 2 and cancer stages III and IV than those without VTE. These patients had a higher mean age, higher mean body mass index (BMI), higher D-dimer level, and lower creatinine clearance (CrCl). The distribution of cancer subtypes, genetic polymorphisms, and other patient characteristics showed similar trends between those with and those without VTE (Table 2).

### Risk factors of VTE at baseline

Table 3 shows the results of the univariable analysis of background factors associated with the risk of VTE at baseline. Although the analysis was univariable, the factors associated with VTE incidence at baseline were age ≥ 65 years (odds ratio [OR] 4.46, 95% confidence interval [CI] 1.70–11.72; *P* = 0.002), ECOG PS of 2 (OR 43.36, 95% CI 8.96–209.76; *P* < 0.001), history of VTE (OR 108.00, 95% CI 9.36– > 999.99; *P* < 0.001), and D-dimer > 1.2 μg/mL (OR 34.64, 95% CI 12.13–98.96; *P* < 0.001). The OR for stage IV and distant metastasis was approximately 2.5, but there was no significant difference.

**Table 3:** Univariable analysis of background factors associated with VTE prevalence at baseline

Factor	*N*	Events, *n* (%)	OR^a^	95% CI	*P*-value
Sex					
Male	3	0 (0.0)	Ref	–	–
Female	990	20 (2.0)	NC	NC	NC
Age, years					
< 65	645	6 (0.9)	Ref	–	–
≥ 65	348	14 (4.0)	4.46	1.70–11.72	0.002
Cancer stage					
II	729	12 (1.6)	Ref	–	–
III	165	4 (2.4)	1.48	0.47–4.66	0.499
IV	99	4 (4.0)	2.52	0.80–7.96	0.116
Lymph node metastasis					
No	510	10 (2.0)	Ref	–	–
Yes	483	10 (2.1)	1.06	0.44–2.56	0.902
Distant metastasis					
No	902	16 (1.8)	Ref	–	–
Yes	91	4 (4.4)	2.55	0.83–7.78	0.101
Occurrence of cancer					
Primary	938	18 (1.9)	Ref	–	–
Recurrence	55	2 (3.6)	1.93	0.44–8.53	0.386
ECOG PS					
0	941	16 (1.7)	Ref	–	–
1	45	1 (2.2)	1.31	0.17–10.13	0.793
2	7	3 (42.9)	43.36	8.96–209.76	< 0.001
BMI, kg/m^2^					
< 25	659	12 (1.8)	Ref	–	–
≥ 25	331	8 (2.4)	1.34	0.54–3.30	0.531
ER					
Negative	235	7 (3.0)	Ref	–	–
Positive	740	13 (1.8)	0.58	0.23–1.48	0.255
PgR					
Negative	381	9 (2.4)	Ref	–	–
Positive	594	11 (1.9)	0.78	0.32–1.90	0.584
HER2					
Negative	711	15 (2.1)	Ref	–	–
Positive^b^	251	5 (2.0)	0.94	0.34–2.62	0.911
History of VTE					
No	990	18 (1.8)	Ref	–	–
Yes	3	2 (66.7)	108.00	9.36– > 999.99	< 0.001
Platelet count, × 10^9^/L					
< 350	889	17 (1.9)	Ref	–	–
≥ 350	87	2 (2.3)	1.21	0.27–5.31	0.804
Hb, g/dL					
≥ 10	953	19 (2.0)	Ref	–	–
< 10	23	0 (0.0)	NC	NC	NC
WBC count, × 10^9^/L					
≤ 11	960	18 (1.9)	Ref	–	–
> 11	16	1 (6.3)	3.49	0.44–27.85	0.238
CrCl, mL/min					
> 50	917	16 (1.7)	Ref	–	–
≤ 50	59	3 (5.1)	3.02	0.85–10.66	0.086
D-dimer, μg/mL					
≤ 1.2	871	5 (0.6)	Ref	–	–
> 1.2	84	14 (16.7)	34.64	12.13–98.96	< 0.001

*BMI* body mass index, *CI* confidence interval, *CrCl* creatinine clearance, *ECOG PS* Eastern Cooperative Oncology Group Performance Status, *ER* estrogen receptor, *Hb* hemoglobin, *HER2* human epidermal growth factor receptor 2, *ICH* immunohistochemistry, *NC* not calculated, *OR* odds ratio, *PgR* progesterone receptor, *Ref* reference value, *VTE* venous thromboembolism, *WBC* white blood cell

^a^Odds ratios were not calculated for categories with 0 events or factors with 0 references

^b^HER2 positive is a composite of ICH3 positive or ICH2 positive with ICH amplification

### Incidence of main outcomes during follow-up

The mean follow-up period was 376.2 days. The incidence of each event during the follow-up period is summarized in Table 4. Among all patients with breast cancer, the incidence of symptomatic VTE was 0.4% (95% CI 0.1–1.0); that of incidental VTE requiring treatment was 0.1% (95% CI 0.0–0.6); composite VTE, 0.5% (95% CI 0.2–1.2); bleeding, 0.2% (95% CI 0.0–0.7); cerebral infarction/TIA/SEE, 0.2% (95% CI 0.0–0.7); and all-cause death, 2.1% (95% CI 1.3–3.2). Of the deaths during the study, one patient who had VTE at baseline developed symptomatic VTE (PE) during the follow-up period and died.

**Table 4:** Incidence of events during the follow-up period

Event	Patients with breast cancer (*n* = 993 [100%])		Patients with VTE at baseline (*n* = 20 [2.0%])		Patients without VTE at baseline (*n* = 973 [98.0%])	
	Patients with events,* n*	Incidence (95% CI)	Patients with events, *n*	Incidence (95% CI)	Patients with events, *n*	Incidence (95% CI)
Symptomatic VTE	4	0.4 (0.1–1.0)	1	5.0 (0.1–24.9)	3	0.3 (0.1–0.9)
Incidental VTE requiring treatment	1	0.1 (0.0–0.6)	0	0.0 (0.0–16.8)	1	0.1 (0.0–0.6)
Composite VTE^a^	5	0.5 (0.2–1.2)	1	5.0 (0.1–24.9)	4	0.4 (0.1–1.0)
Bleeding^b^	2	0.2 (0.0–0.7)	0	0.0 (0.0–16.8)	2	0.2 (0.0–0.7)
Cerebral infarction/TIA/SEE	2	0.2 (0.0–0.7)	0	0.0 (0.0–16.8)	2	0.2 (0.0–0.7)
All-cause death	21	2.1 (1.3–3.2)	1	5.0 (0.1–24.9)	20	2.1 (1.3–3.2)

*CI* confidence interval, *SEE* systemic embolic event, *TIA* transient ischemic attack, *VTE* venous thromboembolism

^a^A composite of symptomatic VTE events and incidental VTE events requiring treatment

^b^Included major bleeding and clinically relevant non-major bleeding events

**Fig. 1:**
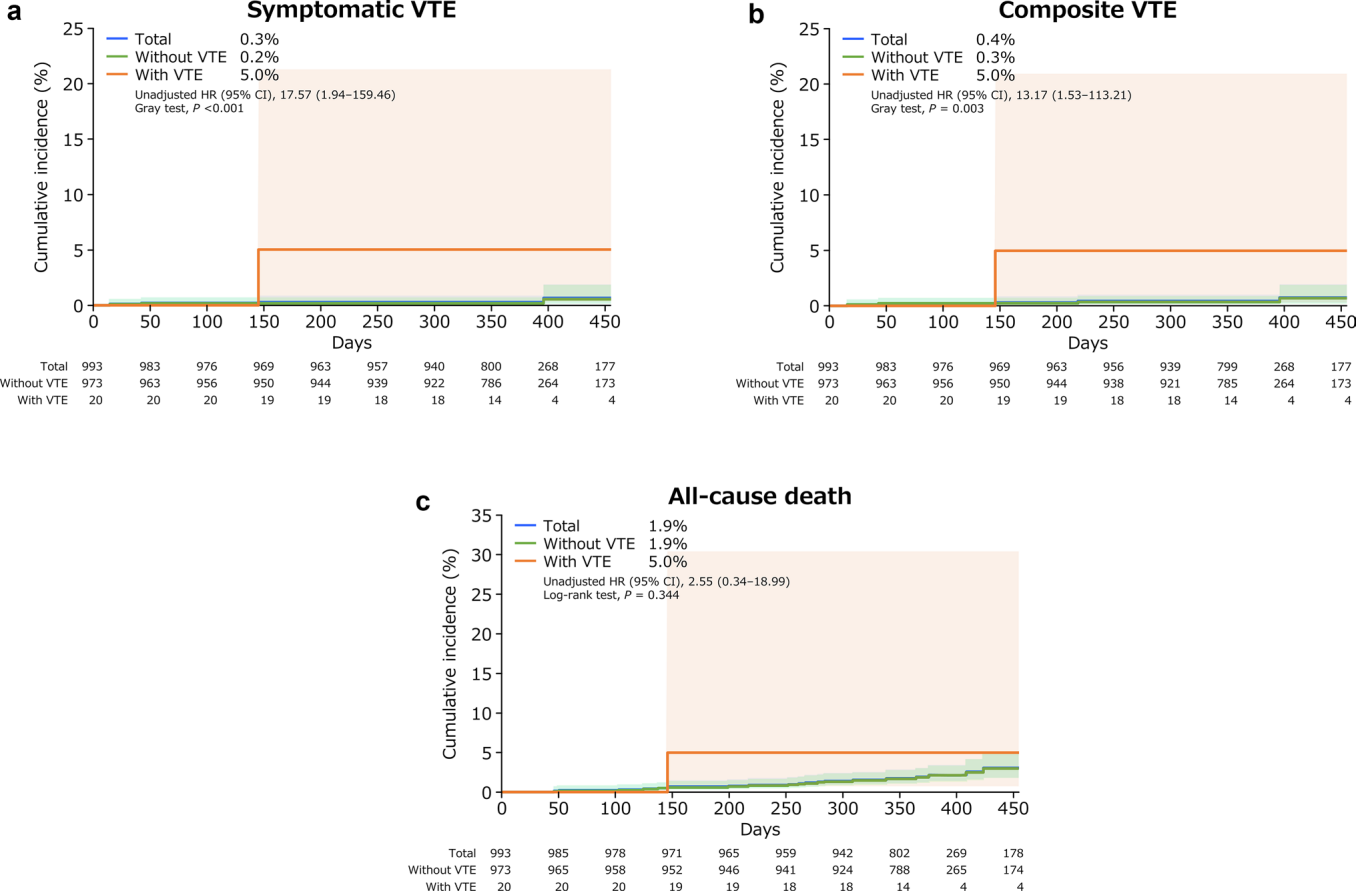
Cumulative incidence of events (time-to-event analysis). **a** symptomatic VTE, **b** composite VTE, and **c** all-cause death. *P* values were calculated using the Gray test (**a**, **b**) or the log-rank test (**c**). Lightly shaded areas represent 95% CIs. *CI* confidence interval, *HR* hazard ratio, *VTE* venous thromboembolism

The cumulative incidences according to VTE at baseline are shown in Fig. 1 and Online Resource 1. Patients with VTE at baseline had a higher hazard ratio (HR) for symptomatic VTE (unadjusted HR 17.57, 95% CI 1.94–159.46; Gray test,* P* < 0.001), and composite VTE (unadjusted HR 13.17, 95% CI 1.53–113.21; Gray test, *P* = 0.003), but there was no significant difference for all-cause death, bleeding, and cerebral infarction/TIA/SEE. Because of the small number of events that occurred, it was impossible to clarify the effect of the presence or absence of VTE at enrollment. No notable trends were identified in the timing of each event.

### Univariable analysis of risk factors for composite VTE during the follow-up period

The univariable analysis of risk factors for composite VTE during the follow-up period is shown in Table 5. During follow-up, the risk factor with significant HR for composite VTE among patients with breast cancer was VTE prevalence at baseline (HR 13.17 95% CI 1.53–113.21;* P* = 0.019). The number of events for patients aged ≥ 65 years, with high BMI (≥ 25 kg/m^2^), high platelet count (≥ 350 × 10^9^/L), and high D-dimer levels (> 1.2 μg/mL) was more than two-fold those for the reference values; however, no statistically significant differences were observed for the respective HRs. There were no significant differences in ER, PgR, or HER2 status.

**Table 5:** Univariable analysis of risk factors for composite VTE during the follow-up period

Factor	*N*	Events, *n* (%)	HR^a^	95% CI	*P* value
Age, years					
< 65	645	2 (0.3)	Ref	–	–
≥ 65	348	3 (0.9)	2.80	0.47–16.64	0.257
Cancer stage					
II	729	4 (0.5)	Ref	–	–
III	165	1 (0.6)	1.09	0.12–9.79	0.939
IV	99	0 (0.0)	NC	NC	NC
ECOG PS					
0	941	5 (0.5)	Ref	–	–
1	45	0 (0.0)	NC	NC	NC
2	7	0 (0.0)	NC	NC	NC
Occurrence of cancer					
Primary	938	5 (0.5)	Ref	–	–
Recurrence	55	0 (0.0)	NC	NC	NC
VTE at baseline					
No	973	4 (0.4)	Ref	–	–
Yes	20	1 (5.0)	13.17	1.53–113.21	0.019
BMI, kg/m^2^					
< 25.0	659	2 (0.3)	Ref	–	–
≥ 25	331	3 (0.9)	3.00	0.50–18.01	0.229
ER					
Negative	235	0 (0.0)	Ref	–	–
Positive	740	5 (0.7)	NC	NC	NC
PgR					
Negative	381	2 (0.5)	Ref	–	–
Positive	594	3 (0.5)	0.96	0.16–5.78	0.967
HER2^b^					
Negative	711	4 (0.6)	Ref	–	–
Positive	251	1 (0.4)	0.75	0.09–6.57	0.793
Platelet count, × 10^9^/L					
< 350	889	4 (0.4)	Ref	–	–
≥ 350	87	1 (1.1)	2.56	0.29–22.76	0.400
Hb, g/dL					
≥ 10	953	5 (0.5)	Ref	–	–
< 10	23	0 (0.0)	NC	NC	NC
WBC count, × 10^9^/L					
≤ 11	960	5 (0.5)	Ref	–	–
> 11	16	0 (0.0)	NC	NC	NC
CrCl, mL/min					
> 50	917	5 (0.5)	Ref	–	–
≤ 50	59	0 (0.0)	NC	NC	NC
D-dimer, μg/mL					
≤ 1.2	871	4 (0.5)	Ref	–	–
> 1.2	84	1 (1.2)	2.61	0.29–23.43	0.392

*BMI* body mass index, *CI* confidence interval, *CrCl* creatinine clearance, *ECOG PS* Eastern Cooperative Oncology Group Performance Status, *ER* estrogen receptor, *Hb* hemoglobin, *HER2* human epidermal growth factor receptor 2, *HR* hazard ratios, *ICH* immunohistochemistry, *NC* not calculated, *PgR* progesterone receptor, *Ref* reference value, *VTE* venous thromboembolism, *WBC* white blood cell

^a^Hazard ratios were not calculated for categories with 0 events or factors with 0 references

^b^HER2 positive is a composite of ICH3 positive or ICH2 positive with ICH amplification

The risk factors of composite VTE during the follow-up period by cancer therapy are shown in Table 6. No significant increase in the number of events was observed for surgery, chemotherapy, or radiotherapy, although adjustments for the patient background characteristics were not performed. There was no significant increase in the risk of composite VTE among patients receiving hormone therapy (HR, 0.21; 95% CI 0.02–1.87,* P* = 0.161). Additionally, among 199 patients (20%) who received tamoxifen, none presented with VTE events during the follow-up period (data not shown).

**Table 6:** Univariable analysis of risk factors for composite VTE during the follow-up period by cancer therapy

Cancer therapy^a^	*N*	Events, *n* (%)	HR	95% CI	*P* value
Surgery					
No	171	1 (0.6)	Ref	–	–
Yes	822	4 (0.5)	0.84	0.09–7.68	0.881
Chemotherapy					
No	63	1 (1.6)	Ref	–	–
Yes	930	4 (0.4)	0.27	0.03–2.39	0.237
Hormone therapy^b^					
No	458	4 (0.9)	Ref	–	–
Yes	535	1 (0.2)	0.21	0.02–1.87	0.161
Radiation therapy					
No	562	4 (0.7)	Ref	–	–
Yes	431	1 (0.2)	0.31	0.04–2.70	0.291

*CI* confidence interval, *HR* hazard ratio, *LH-RH* luteinizing hormone-releasing hormone, *Ref* reference value, *VTE* venous thromboembolism

^a^Cancer therapy performed after the onset date of the composite VTE event was considered as no treatment

^b^Patients treated either with LH-RH agonists, tamoxifen, anastrozole, letrozole, or exemestane

## Discussion

This was a subgroup analysis of the Cancer-VTE Registry, a large nationwide registry with a 1-year follow-up that enrolled about 10,000 patients with six different solid tumor types [3]. This subgroup analysis focused on evaluating VTE occurrence among patients with breast cancer and identifying background factors that can increase the risk of VTE in this population. These data were previously scarce but are relevant as the incidence of breast cancer has been growing in Japan and other Asian countries in recent years [22].

The study reported a significantly higher incidence of symptomatic VTE in the follow-up period in patients with VTE at enrollment than those without [3]. Patients with breast cancer with VTE at enrollment also had a higher incidence of both symptomatic and composite VTE. As with the results of this study, previous studies have reported that pre-existing VTE is a risk factor for VTE events during cancer treatment in patients with breast cancer [11].

Compared with the overall baseline results of the Cancer-VTE Registry [3], patients with breast cancer had a lower VTE prevalence (2.0% vs 5.9%), which was consistent with previous studies [13]. In the Cancer-VTE Registry, patients with breast cancer were younger than patients with other cancer types [3]. Moreover, patients with breast cancer and metastatic disease at the time of diagnosis were reported to have a five-fold increased risk of developing VTE compared with those with localized disease [23, 24], but among patients with breast cancer in the Cancer-VTE Registry, there were few patients with advanced cancer, and stage II accounted for more than 70% of patients with breast cancer. There was no significant difference by stage, but the baseline VTE prevalence was 2.5 times higher in stage IV patients than in stage II patients (OR 2.52, 95% CI 0.80–7.96; *P* = 0.116), suggesting that, if we focus only on advanced breast cancer patients, it can be assumed that the VTE incidence would be several times higher than 2.0%. Furthermore, overweight is thought to be a risk factor of VTE, but the number of overweight patients was quite small (Table 2), so it was not detected at a significant level.

Conversely, the post-discharge VTE frequency in surgically treated patients with breast cancer was reported to be 0.0–0.8% [25, 26], with similar results found in the present study. The incidence of VTE was higher than that in the general population, as in a previous study in non-Japanese patients [11]. Based on the above, we cannot conclude that the risk of VTE in all patients with breast cancer is lower than that of other cancer types.

Among patients with breast cancer, risk factors for VTE at baseline were age ≥ 65 years, ECOG PS of 2, a history of VTE, and D-dimer > 1.2 μg/mL, and these risk factors were similar to those reported previously in other populations of patients with breast cancer (i.e., older age, high BMI, pre-existing VTE, comorbid disease, cancer subtype, tumor size and metastasis, PgR-negative status, and treatment with chemotherapy and hormone therapy) [11, 27].

Of note, in this study, all 20 patients with breast cancer and VTE at baseline had asymptomatic DVT. Previous studies have reported a high risk of recurrence of cancer-related VTE among patients with cancer-related asymptomatic distal DVT [28, 29]. Another previous study reported that the incidence of fatal PE in patients with breast cancer was 2.4%, which is higher than that of other carcinomas [30]. In fact, one of the 20 patients with VTE at enrollment developed PE during the observation period, and the outcome of this event was death in the present study. Thus, it may be advisable to measure D-dimer in patients with breast cancer at high risk for VTE. Especially in breast cancer patients with a high level of D-dimer, venous ultrasonography of the lower extremities may be a better option.

By subgroup, there were no major differences in the event occurrence by cancer subtype or hormone receptor status. Each hormone receptor status is associated with different therapeutics and patient backgrounds, which may influence events such as VTE. However, this study did not account for these effects. Hormone therapy is the primary drug treatment for hormone receptor-positive breast cancer but has been reported as a treatment-related risk factor for VTE in cancer patients [16, 31, 32]. An underlying mechanism could be the increased coagulability associated with tamoxifen [33]. More than half (535/993) of the patients in this study also received hormone therapy, and although VTE events occurred less frequently with hormone therapy than without (HR 0.21, 95% CI 0.02–1.87;* P* = 0.161), it was consistent with previous studies reporting that hormone therapy was not as significant a risk factor [7, 34, 35]. In contrast, some studies reported that the concomitant use of some anticancer drugs and hormone therapy increases the VTE risk among hormone-treated patients with breast cancer [36]. In the present study, this may have been due to the lack of background adjustment for patients with and without hormone therapy, as well as the short observation period of 1 year.

### Limitations

The main limitations of the Cancer-VTE Registry have been previously reported [3, 18]. The multivariable analysis for identifying risk factors could not be performed because there were few events. For the same reason, it should be noted that wide ranges were observed in the 95% CIs of the univariable analysis results, limiting their interpretation. Hormone therapy is given over an extended period; thus, follow-up during 1 year of cancer treatment may have been insufficient to assess the effect of hormone therapy on VTE development. The distributions of stages II, III, and IV in this study are similar to those reported by the Japanese Breast Cancer Registry [37], and we believe these results can be generalized to the overall population. However, the number of registered Stage IV patients at high risk of VTE was small, and this was insufficient to clarify the actual status of VTE in advanced breast cancer patients at high risk of VTE. We believe further studies with targeted designs need to be conducted to clarify VTE risk in patients with advanced breast cancer.

## Conclusions

The Cancer-VTE Registry, a large-scale prospective observational study, revealed the actual status of VTE incidences in Japanese patients with breast cancer. In this subgroup analysis, over 70% of patients had stage II breast cancer, and 2.0% had VTE, as revealed by VTE screening before the start of cancer treatment. Although the incidence of VTE was also low (symptomatic VTE 0.4%, incidental VTE requiring treatment 0.1%) during the follow-up period, one patient had a fatal outcome due to PE.

## Supplementary Information

Below is the link to the electronic supplementary material.

Supplementary file1 Online Resource 1. Cumulative incidence of events (time-to-event analysis). (A) Bleeding and (B) cerebral infarction/TIA/SEE. P-values were calculated using the Gray test. Lightly shaded areas represent 95% CIs. CI, confidence interval; HR, hazard ratio; SEE, systemic embolic event; TIA, transient ischemic attack; VTE, venous thromboembolism (TIF 599 KB)

Supplementary file2 (TIF 612 KB)

## Data Availability

The anonymized data underlying the results presented in this manuscript may be made available to researchers upon submission of a reasonable request to the corresponding author. The decision to disclose the data will be made by the corresponding author and the funder, Daiichi Sankyo Co., Ltd. The data disclosure can be requested for 36 months from the article publication.

## Abbreviations

BMI: Body mass index
CI: Confidence interval
CrCl: Creatinine clearance
DVT: Deep vein thrombosis
ECOG PS: Eastern Cooperative Oncology Group Performance Status
ER: Estrogen receptor
HR: Hazard ratio
OR: Odds ratio
PE: Pulmonary embolism
PgR: Progesterone receptor
SEE: Systemic embolic event
TIA: Transient ischemic attack
VTE: Venous thromboembolism
