# Supplementary figures and images for: Venous thromboembolism in Japanese patients with breast cancer: subgroup analysis of the Cancer-VTE Registry
Source: Breast Cancer. 2023 Apr 17;30(4):607–16. doi: 10.1007/s12282-023-01452-7 (PMC10284999; doi:10.1007/s12282-023-01452-7)

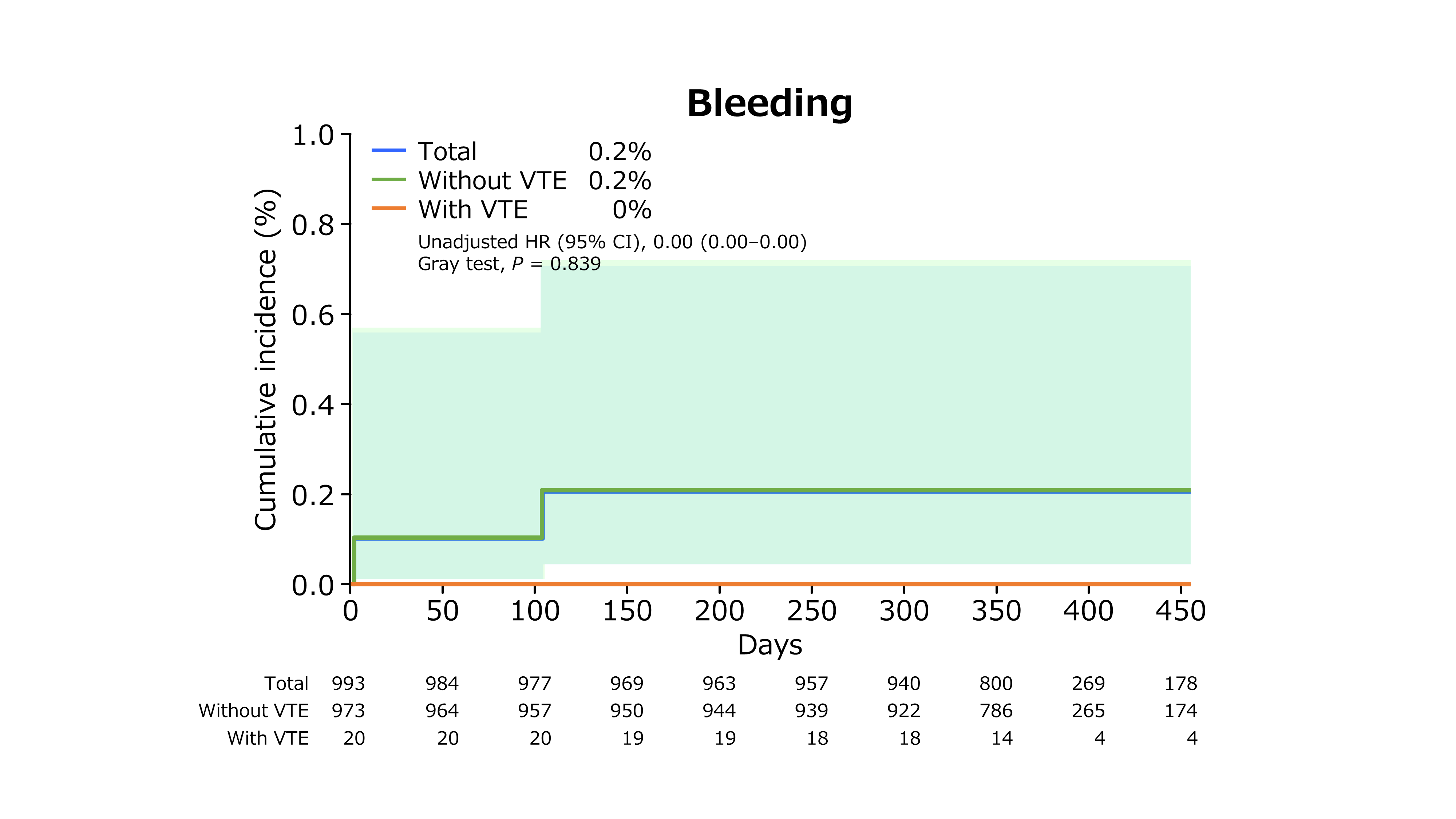

Supplement: Supplementary file 1 — Supplementary file1 Online Resource 1. Cumulative incidence of events (time-to-event analysis). (A) Bleeding and (B) cerebral infarction/TIA/SEE. P-values were calculated using the Gray test. Lightly shaded areas represent 95% CIs. CI, confidence interval; HR, hazard ratio; SEE, systemic embolic event; TIA, transient ischemic attack; VTE, venous thromboembolism (TIF 599 KB) [file 12282_2023_1452_MOESM1_ESM.tif]

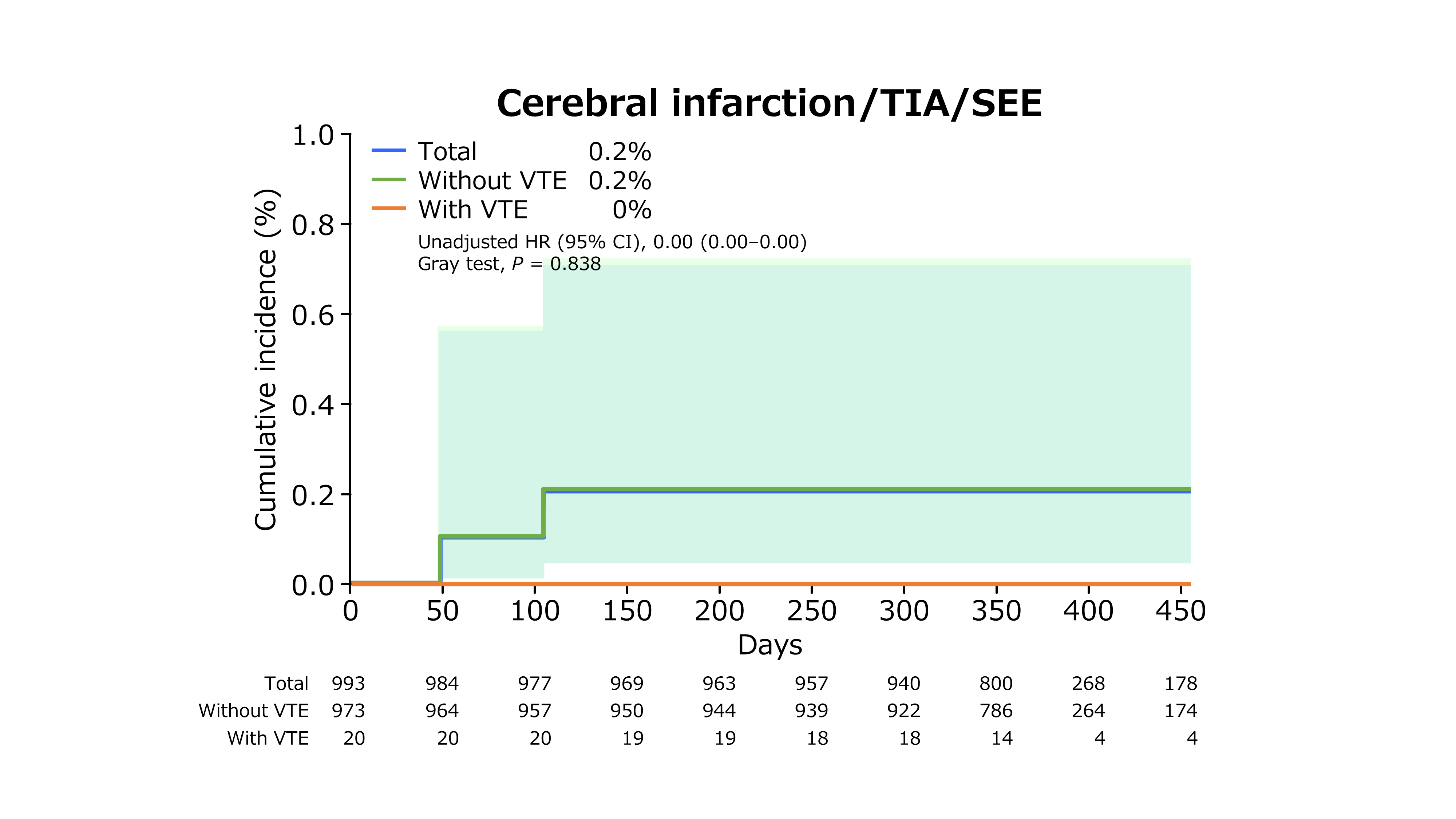

Supplement: Supplementary file 2 — Supplementary file2 (TIF 612 KB) [file 12282_2023_1452_MOESM2_ESM.tif]
